# Tipping the balance toward transplantation tolerance: in vivo therapy using a mutated IL-2

**DOI:** 10.1172/JCI178570

**Published:** 2024-03-01

**Authors:** Geoffrey Camirand, Fadi G. Lakkis

**Affiliations:** The Thomas E. Starzl Transplantation Institute, Departments of Surgery and Immunology, University of Pittsburgh School of Medicine, Pittsburgh, Pennsylvania, USA.

## Abstract

Immune tolerance to allogenic transplanted tissues remains elusive, and therapeutics promoting CD4^+^FOXP3^+^ Tregs are required to achieve this ultimate goal. In this issue of the *JCI*, Efe and colleagues engineered an Fc domain fused to a human mutein IL-2 (mIL-2–Fc) bearing mutations that confer preferential binding to the high-affinity IL-2 receptor expressed on Tregs. In vivo mIL-2–Fc therapy effectively heightened mouse, monkey, and human Treg numbers, promoted tolerance to minor antigen mismatched skin grafts in mice, and synergized with immunosuppressive drugs used in the clinic. These findings warrant clinical trials that assess the efficacy of mIL-2–Fc in transplantation.

## Engineering altered IL-2 protein to control immunity

The IL-2 cytokine is key in controlling immunity, as it can both promote and suppress immunity depending on which immune cell it signals through. IL-2 can bind to the intermediate-affinity dimeric receptor, which is composed of CD122 (IL-2Rβ) and CD132 (IL-2Rγ), or to the high-affinity trimeric receptor composed of CD25 (IL-2Rα), CD122, and CD132. The dimeric IL-2R is mainly found on memory T cells and NK cells, whereas the trimeric IL-2 receptor is found on CD4^+^FOXP3^+^ Tregs, on recently activated CD4^+^ and CD8^+^ effector T cells, on some NK cells and NKT cells, and on group 2 innate lymphoid cells (ILC2s) (1). Treg homeostasis critically relies on IL-2R signaling, and Tregs dramatically expand upon IL-2R signaling. Thus, given the divergent expression of these various IL-2 receptors (IL-2Rs), IL-2 therapy has been an attractive therapeutic concept to promote either immunity (by triggering the intermediate-affinity dimeric IL-2R on effector immune cells to combat cancer), or immune suppression (by triggering the high-affinity trimeric IL-2R on Tregs in the context of autoimmunity and transplantation).

However, given the double-edged sword aspect of IL-2 therapy, its use in the clinic can lead to unexpected outcomes. One example includes a clinical trial in which stable liver transplant recipients received low-dose IL-2 as a means to promote Tregs and reduce immunosuppression (2). In preclinical studies, low-dose IL-2 was shown to preferentially expand Tregs over effector T cells and NK cells (3). All of the patients with liver transplants who received low-dose IL-2 had elevated circulating Tregs. However, low-dose IL-2 in this clinical trial was detrimental, as the patients developed signs of rejection, leading to an early termination of the trial (2). Similar observations were reported in a clinical trial involving patients with type 1 diabetes who concomitantly received low-dose IL-2 and Treg therapy (4). Low-dose IL-2 treatment not only effectively promoted both adoptively transferred and endogenous Tregs, but also expanded cytotoxic immune cells.

On the other hand, low-dose IL2 therapy has demonstrated beneficial effects in some clinical trials for the treatment of various autoimmune disorders, more specifically in patients with systemic lupus erythematosus (5). Thus, it appears that the outcomes under low-dose IL-2 therapy may vary depending on the underlying cytotoxic immune response at the time of IL-2 treatment, with the parallel Treg expansion having an insufficient inhibitory effect on these cytotoxic immune cells. Overall, these trials demonstrate that improved therapeutics targeting specific IL-2Rs are necessary.

Consequently, efforts have been expended to enhance the specificity of IL-2R targeting and to increase the bioavailability of IL-2. To these ends, several engineering approaches were undertaken: IL-2 muteins, PEGylated IL-2, IL-2–anti–IL-2 immune complexes and IL-2–Fc, and IL-2–CD25 fusion proteins (6).

IL-2 muteins were developed to skew the specificity of IL-2 toward either the high-affinity trimeric or the intermediate-affinity dimeric IL-2R. To achieve such a goal, these mutated IL-2 proteins displayed a reduced affinity either for CD122 or CD25. The crystal structure of IL-2 bound to the IL-2R was helpful in identifying the amino acid residues implicated in IL-2 interactions with either CD122 or CD25. Consequently, targeted amino acid substitutions led to two versions of IL-2 mutein (no-α IL-2 and IL-2 superkine) displaying lower affinity for CD25 and thus favored dimeric IL-2R signaling on memory T cells and NK cells. In vivo therapy using either no-α IL-2 or IL-2 superkine enhanced immune responses to tumors, owing to increased CD8^+^ T cell responses and lower Treg expansion compared with native IL-2 therapy. IL-2 muteins with lower affinity for CD122, and thus preferential specificity for the trimeric IL-2R, were also generated [called IgG–(IL-2N88D)_2_ and Fc.Mut24]. Although these muteins displayed a skewed affinity for CD25 over CD122, their affinity for CD25 was reduced compared with native IL-2–Fc, which is an important aspect that was avoided with the IL-2 mutein presented in this issue of the *JCI* by Efe and colleagues (7). Despite their lower affinities for CD25, in vivo treatment with either IgG–(IL-2N88D)_2_ or Fc.Mut24 promoted Tregs and protected from diabetes and graft-versus-host disease (6). However, these Treg-promoting muteins have not been assessed in transplantation settings.

## mIL-2–Fc promotes tolerance to nominal graft antigen

Approaches that promote endogenous Tregs provide attractive advantages over adoptive transfer of exogenous Tregs, which are proving costly and time consuming. Given that the field of transplantation is in dire need of new therapeutics that promote Treg-dependent tolerance, the trimeric high-affinity IL-2R represents an appealing therapeutic target to achieve this goal.

To this end, Efe and collaborators designed an IL-2 mutein fused to the Fc portion of a human IgG1 antibody (termed mIL-2–Fc) (7). Histidine 16 on native IL-2 is structurally essential for the interaction of IL-2 with CD122. Hence, histidine 16 was substituted with a hydrophobic leucine residue. This substitution resulted in a lower affinity of mIL-2–Fc for the dimeric CD122/CD132 IL-2R, while preserving near-native affinity for the trimeric CD25/CD122/CD132 IL-2R. mIL-2–Fc demonstrated a half-life of approximately 9 hours in mice, with nominal detection at 72 hours. Importantly, mIL-2–Fc exhibited specificity for Tregs obtained from either mice, monkeys, or humans. mIL-2–Fc induced downstream IL-2R signaling in both mouse and human Tregs in vitro. In addition, mIL-2–Fc given to mice or monkeys caused a drastic 4-fold increase in Tregs, with nominal changes to CD8^+^ T cells and NK cells compared with the control. Notably, the increase in Treg numbers could be sustained with continuous mIL-2–Fc treatment over a three-week period, with Treg numbers returning to baseline levels upon cessation of mIL-2–Fc treatment.

**Figure 1:**
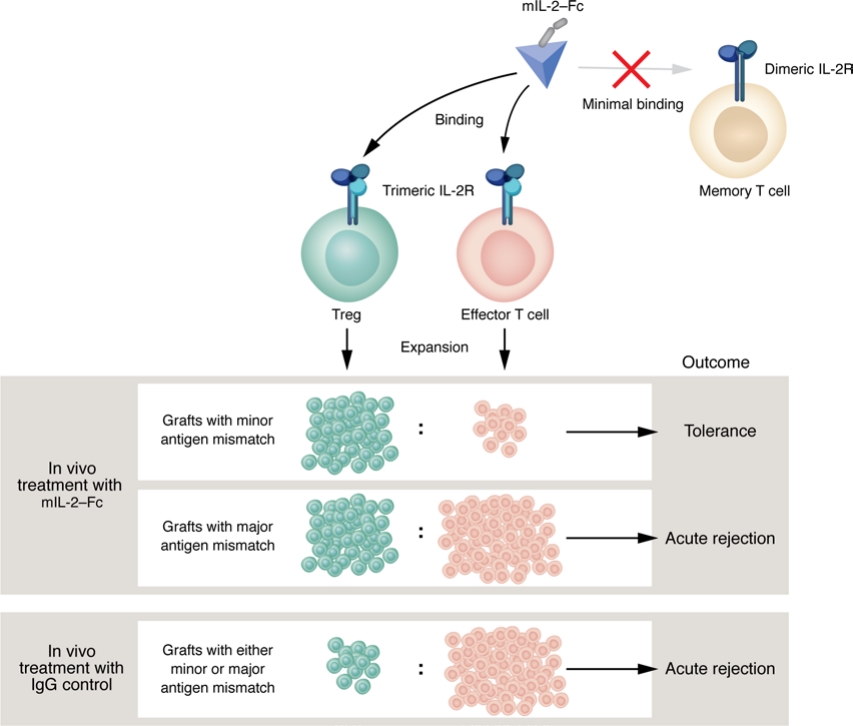
mIL-2–Fc with trimeric IL-2R specificity induces tolerance in low effector T cell contexts. The high-affinity trimeric IL-2R is expressed on Tregs and on recently activated effector T cells among others. mIL-2–Fc with specificity for the trimeric IL-2R, but not the dimeric IL-2R expressed on memory T cells and NK cells, expands Tregs in vivo and induces immunological tolerance to minor antigen-mismatched skin grafts in which low effector T cell levels are present at the time of treatment. In contrast, immunological tolerance with mIL-2–Fc treatment cannot be achieved against major antigen-mismatched skin grafts in which elevated effector T cell levels are present at the time of treatment, despite expansion of Tregs.

The expanded Tregs under mIL-2–Fc treatment demonstrated all the hallmarks of effector Tregs, which are a highly activated and actively dividing Treg subset. They also showed an enhanced suppressive capacity in ex vivo suppression assays. Crucially, a sustained mIL-2–Fc regimen alone was able to provide protection from rejection of minor antigen-mismatched skin grafts, with approximately 60% to 75% of the grafts surviving several weeks after cessation of mIL-2–Fc treatment. Interestingly, the mice bearing long-term surviving skin grafts demonstrated donor-specific tolerance, suggesting that mIL-2–Fc promoted immune regulation to donor antigens (Figure 1). Consistently, early histological analysis of primary skin grafts from mIL-2–Fc–treated mice showed elevated Treg to effector T cell ratios compared with controls.

In contrast to these results, the same sustained mIL-2–Fc regimen alone failed to protect major antigen-mismatched skin grafts from rejection, with all grafts rejected at the same tempo as that seen in control untreated mice. This outcome occurred despite elevated numbers of circulating Tregs. However, mIL-2–Fc treatment synergized with a commonly used immunosuppressive drug, tacrolimus, and delayed the rejection of skin allografts compared with individual treatments. One can assume that additional immunosuppression is necessary in this context to control the strong and rapid effector T response to major or multiple alloantigens, which does not occur under minor antigen-mismatched skin grafts.

Interestingly, this lack of protection of major antigen-mismatched skin grafts with mIL-2–Fc treatment alone was also observed in a previous study using IL-2/anti–IL-2 complexes that target all IL-2Rs (8). In addition, treatment with the IL-2/anti–IL-2 complexes synergized with immunosuppression, as similarly observed with mIL-2–Fc treatment. These strikingly similar results raise the question as to whether mIL-2–Fc provides a therapeutic advantage in the context of a strong effector T cell response over the treatment with native IL-2, or with other IL-2 muteins that promote Tregs. Further studies are warranted to answer these questions. In addition, it remains unknown whether mIL-2–Fc also promoted recently activated CD25^+^ effector T cells, which would explain the lack of a protective effect in major antigen-mismatched skin graft recipients when used as a monotherapy.

## Perspectives and conclusions

Efe and collaborators (7) present crucial and encouraging data on the efficacy of a Treg-promoting IL-2 mutein with low affinity for CD122 and native affinity for CD25 in the context of transplantation. Their report, combined with our current knowledge, highlights outcomes and considerations for the use of available IL-2 muteins that promote signaling through the trimeric IL-2R, including (a) that a robust antigen-specific tolerance can be achieved; (b) the underlying cytotoxic immune context in which these IL-2 muteins are used dictates outcomes toward either immunity in high cytotoxicity contexts, or immune suppression in low cytotoxicity contexts (Figure 1); and (c) immunosuppressive drugs controlling the underlying cytotoxic immune response synergize with IL-2 mutein to improve graft survival outcomes. Thus, future clinical trials assessing the therapeutic efficacy of IL-2 muteins in transplantation will need to be carefully designed to avoid their use during elevated cytotoxic immune circumstances. In addition, further engineering efforts that enhance the specificity of IL-2 muteins for Tregs would greatly improve therapeutic efficacy.

## Author contributions

GC and FGL contributed equally to this Commentary. The order for listing their names was chosen at random.

## Version 1 03/01/2024

Electronic publication

## References

[1] Spolski R (2018). Biology and regulation of IL-2: from molecular mechanisms to human therapy. Nat Rev Immunol.

[2] Lim TY (2023). Low dose interleukin-2 selectively expands circulating regulatory T cells but fails to promote liver allograft tolerance in humans. J Hepatol.

[3] Yuan X (2014). The importance of regulatory T-cell heterogeneity in maintaining self-tolerance. Immunol Rev.

[4] Dong S (2021). The effect of low-dose IL-2 and Treg adoptive cell therapy in patients with type 1 diabetes. JCI Insight.

[5] Graßhoff H (2021). Low-dose IL-2 therapy in autoimmune and rheumatic diseases. Front Immunol.

[6] Hernandez R (2022). Engineering IL-2 for immunotherapy of autoimmunity and cancer. Nat Rev Immunol.

[7] Efe O (2024). A humanized IL-2 mutein expands Tregs and prolongs transplant survival in preclinical models. J Clin Invest.

[8] Pilat N (2019). Treg-mediated prolonged survival of skin allografts without immunosuppression. Proc Natl Acad Sci U S A.

